# Prediction-Correction Techniques to Support Sensor Interoperability in Industry 4.0 Systems

**DOI:** 10.3390/s21217301

**Published:** 2021-11-02

**Authors:** Borja Bordel, Ramón Alcarria, Tomás Robles

**Affiliations:** 1 Information Systems Department, Information Systems School, Campus Sur, Universidad Politécnica de Madrid, 28031 Madrid, Spain; borja.bordel@upm.es (B.B.); tomas.robles@upm.es (T.R.); 2 Department of Geospatial Engineering, School of Surveying Engineering, Campus Sur, Universidad Politécnica de Madrid, 28031 Madrid, Spain

**Keywords:** Industry 4.0, interpolation techniques, predictor-corrector algorithms, data series, sensor nodes

## Abstract

Industry 4.0 is envisioned to transform the entire economical ecosystem by the inclusion of new paradigms, such as cyber-physical systems or artificial intelligence, into the production systems and solutions. One of the main benefits of this revolution is the increase in the production systems’ efficiency, thanks to real-time algorithms and automatic decision-making mechanisms. However, at the software level, these innovative algorithms are very sensitive to the quality of received data. Common malfunctions in sensor nodes, such as delays, numerical errors, corrupted data or inactivity periods, may cause a critical problem if an inadequate decision is made based on those data. Many systems remove this risk by seamlessly integrating the sensor nodes and the high-level components, but this situation substantially reduces the impact of the Industry 4.0 paradigm and increases its deployment cost. Therefore, new solutions that guarantee the interoperability of all sensors with the software elements in Industry 4.0 solutions are needed. In this paper, we propose a solution based on numerical algorithms following a predictor-corrector architecture. Using a combination of techniques, such as Lagrange polynomial and Hermite interpolation, data series may be adapted to the requirements of Industry 4.0 software algorithms. Series may be expanded, contracted or completed using predicted samples, which are later updated and corrected using the real information (if received). Results show the proposed solution works in real time, increases the quality of data series in a relevant way and reduces the error probability in Industry 4.0 systems.

## 1. Introduction

The strengthening of important global crises, such as the climatic crisis or the natural resource crisis, makes essential a change in the productive schemes of all countries, but especially in those with a relevant industrial sector [1]. The increase of efficiency in all industrial production processes is the only solution to optimize the use of resources, support the citizens’ wellbeing and strengthen social development [2]. Industry 4.0 is an innovative paradigm referring to this new era [3].

In Industry 4.0, production systems and solutions implement mechanisms to make flexible, automatic and real-time decisions [4] that guarantee the adaptation of production processes to the variable behavior of economic, social and physical contexts [5]. With this approach, the global efficiency of industry has proved to increase significantly [6]. Paradigms, such as cyber-physical systems [7] or artificial intelligence [8], are basic to enable this new era, although all these monitoring mechanisms and decision-making algorithms are supported by a common technology: sensor nodes [9].

Using the sensor data, high-level software modules may create models to represent (and later predict) the production processes’ and industry context’s evolution, make real-time business decisions and (even) trigger alarms about workers’ safety and wellbeing [10]. Some of these activities are critical in industry, and a precise evaluation and stable, high-quality, physical monitoring are essential to avoid fatal problems. At the software level, algorithms commonly match these requirements. However, sensor nodes are much less stable, and they present many random malfunctions [11].

Sensing platforms may be affected by human actions (including hits, blurs, misalignments, etc.), numerical errors (especially in digital sensors measuring derivate variables, such as the electrical power), hardware problems (such as overheating, aging, inactive periods, etc.), embedded software issues (for example, memory congestion, blocking instructions, etc.) and communication malfunctions (such as variables delays, jitter, packet losses, interferences, etc.), among many other potential impacts [12]. Although all these malfunctions are not relevant if long-term analyses are performed (e.g., for statistical applications), they may cause a critical situation if an inadequate decision is made in real time based on a low-quality data flow. Currently, the way in which this problem is commonly addressed is by considering a seamless integration of sensor nodes and high-level software components [13], integrating calibration phases and algorithms for adjusting or compensating effects, such as delays, jitter or numerical errors, etc.

Nevertheless, this seamless integration highly affects the social and economic impact of the Industry 4.0 paradigm, as only some (or even only one) sensor technologies may be employed in each application, as calibration models, compensation algorithms, etc. [14], are totally dependent on the specific sensors and processing algorithms to be integrated. This turns Industry 4.0 into a very rigid and close paradigm, more similar to a proprietary solution than to a flexible, open approach. Thus, the adoption of the Industry 4.0 paradigm may get very slow because of its high cost (caused by a lack of competitiveness in the market) and its low interoperability.

To address this challenging situation, new solutions guaranteeing the interoperability of all sensor nodes and sensor technologies with every possible software element and processing algorithm in Industry 4.0 solutions are needed.

Therefore, in this paper, we propose a general solution to ensure the high quality of data flows and their adaptation to the algorithms’ requirements, valid for all sensor technologies and algorithms. It is a flexible and adaptable solution that may be integrated in every Industry 4.0 system. The proposed solution is based on numerical algorithms following a predictor-corrector architecture. Given a data flow to be curated and adapted, first, using a combination of techniques, such as Lagrange polynomial and Hermite interpolation, a set of potential, curated data series is calculated. Later, the most probable series is selected according to the statistical properties of the historical series. The original data series may be expanded, contracted or completed using predicted samples, which are later updated and corrected (second phase) using the real information (if received).

Contrary to other proposals (based, for example, on Gaussian distributions), this scheme is not dependent on the sensor technology or the software modules to be integrated, and it introduces (as shown in Section 4) a negligible delay, so real-time operation is not affected (something essential in Industry 4.0 systems). As a result, the sensor platform may interoperate with any high-level application, with no adaptation or calibration procedure. The produced curated time series has enough quality to be integrated with any kind of software module.

The structure of the paper is as follows: Section 2 presents the state-of-the-art on sensor interoperability in Industry 4.0 scenarios. Section 3 describes the main proposal, including the mathematical foundations. Section 4 includes an experimental validation analyzing the performance of the proposed solution. Finally, Section 5 shows the conclusions and future work.

## 2. State of the Art on Sensor Interoperability in Industry 4.0 Scenarios

In the last 10 years, the idea of “sensor interoperability” has been understood in many ways [15]. From some approaches focused on hardware compatibility [16], to other views related to the cyber-physical system revolution and focused on abstract and high-level issues, such as synchronization [17]. However, the most modern and accepted definition for sensor interoperability was proposed by the Institute of Electrical and Electronic Engineers (IEEE) [18]: “the ability of two or more systems or components to exchange information and to use the information that has been exchanged”.

In this context, two basic aspects are identified within the challenge of sensor interoperability: the interconnection technologies and the adaptation technologies [19]. The first ones are focused on enabling the exchange of information, while the second ones aim to allow the use of the exchanged data.

One of the basic contributions to interconnection technologies is interoperability standards and architectures. The Industrial Internet Reference Architecture (IIRA) [20] was introduced in 2015 and provides a design process to integrate interoperability mechanisms in industrial Internet systems. On the contrary, the reference architecture model for Industry 4.0 [21] (also proposed in 2015) includes a set of guidelines to understand previously existing generic interoperability standards in the context of Industry 4.0. Nevertheless, the main topic investigated within this topic is cloud and edge architectures [22]. In cloud architectures, the interoperability problem is decomposed into elemental problems that are solved and executed in a distributed manner [23]. Although these architectures are very flexible and can be employed in all kinds of Industry 4.0 scenarios [24], they implement a star topology where all transactions must go through the cloud, which may cause bottlenecks and congestion under some circumstances [25]. Contrary to these traditional cloud architectures, the proposed solution may be adapted to different Industry 4.0 architectures. It can be deployed in edge architectures, just distributing all the independent modules among the different devices. But the proposed framework also allows several orchestrated instances working in one unique scenario, as the statistical model does not need a global understanding of the physical platform. Then, a mesh architecture can also be supported, where bottlenecks and congestion are easier to manage.

Other and heterogenous interconnection technologies have been also reported. Some proposals define models to fill the gap between low-level infrastructures and data analytics components [26], while other schemes integrate semantic web components and ontologies to connect cyber-physical systems and knowledge management modules [27]. Among all these solutions, digital twinning is the most promising approach [28]. Digital twins are comprehensive digital representations of physical components [29], so they can simulate the behavior of real underlying platforms through realistic models [30]. Using these twins, different enhanced interconnection middleware (for example, based on publication/subscription networks) have been tested in several application scenarios [31]. Although these mechanisms successfully interconnect all layers in an Industry 4.0 system, they cannot protect the high-level applications from errors or corrupted data in the infrastructure. Adaptation technologies are then required.

Regarding adaptation schemes, although data management and processing are one of the most popular, challenging and interesting topics currently [32], most reported Industry 4.0 solutions in this area are focused on high-level applications: from fault diagnosis [33], fault prediction [34] or prognosis [35] to semantic mechanisms [36] or data-fusion approaches [34,37]. However, in all these proposals, sensing data are considered to show the required quality, and no data curation or quality improvement mechanisms are described.

One of the key challenges addressed in the context of adaptation technologies is sensor reliability [38]. In autonomous systems, such as cyber-physical systems, problems, such as lost-data packages and data collision, must be addressed [39]. In general, however, proposed solutions are not focused on data curation but on analyzing how reliable the received data are. Different models to estimate the sensor reliability at any time have been reported: from the traditional, specific models for each sensor (typically motion sensors) [40] and probabilistic graphs [41], to modern machine-learning frameworks [42] or response filters [43] that operate with generic devices. The final objective of all these models is to enrich the decision process based on the collected data. Although promising results have been reported, this approach needs the high-level, decision-making modules to be adapted as well, so the Industry 4.0 implantation costs and barriers tend to be higher. With the proposed solution in this paper, this challenge is addressed.

In semantic architectures, data adaptation is also critical. Different mechanisms to adapt and transform the different semantic standards into any other data format may be found [44]. Besides, ontologies to allow semantic data processing have been reported [45]. Contrary to the proposed solution, these schemes cannot be employed to protect the Industry 4.0 system against corrupted data, malfunctions, etc.

On the other hand, data-curation mechanisms are not explicitly addressed, as a seamless integration among hardware and software components [34,46] is the preferred approach in the literature. Hard and complex calibration processes are usually considered [6] to make the processing algorithms aware of the sensor nodes’ biases. Besides, computationally heavy schemes to compensate different effects (such as redundant data) based on previous observations and offline processing may be found [47]. However, all these proposals do not enable sensor interoperability (they are totally application-specific); on the contrary, they make it difficult. Moreover, they are not flexible or dynamic solutions and, of course, they cannot be executed in real time (essential requirements in Industry 4.0).

Only a few proposals on actual data curation have been reported. In this area, most contributions are focused on outlier detection [11]. Using different techniques, datasets are transformed, and anomalous data are removed. Techniques based on digital encoders [48], machine learning [49], statistical indicators [50], performance indicators [16] or hybrid approaches [51] have been described. Although these schemes are useful, they cannot be employed in real time, and many other potential malfunctions, such as packet losses, cannot be addressed through these solutions. On the other hand, mechanisms based on signal-processing techniques may be found [15]. In these solutions, data are understood as communication signals, and they are curated based on instruments, such as the complex envelope. This approach may operate in real time and may correct and curate all kinds of malfunctions; however, it only considers one criterion to propose a curated data series. Thus, the error introduced by the curation algorithm is very variable, depending on how similar the sensor data under curation to a communication signal is. In some Industry 4.0 scenarios, this error may be too high to be acceptable.

Finally, some generic proposals on data analysis may be employed to support data curation in Industry 4.0. For example, algorithms to classify time series in an automatic and more flexible manner [52] have been reported. If only two labels (valid and invalid) are defined, this scheme could be employed for data curation. However, it cannot be employed in real time, and it does not enable the correction of errors in data series.

Contrary to all these previous proposals, the solution described in this paper may operate in real time, as it only operates with a limited amount of data. It is flexible and adaptable to all scenarios, as it does not depend on the sensor technology of software algorithms to be employed. Besides, all kinds of malfunctions can be curated, and up to four different potential curated data series are analyzed before selecting the most probable one.

## 3. Proposed Predictor-Corrector Solution

In this section, the proposed data duration mechanism, based on a predictor-corrector approach, is presented. Section 3.1 describes the general statistical framework to calculate and obtain the curated data series. Section 3.2 presents the different approaches to calculate the actual curated data flows, even in real time. Section 3.3 describes the models to analyze and estimate the different data malfunctions that may appear in Industry 4.0 solutions. Finally, Section 3.4 presents the mechanisms to update the curated data series if real data from Industry 4.0 is received in the future.

### 3.1. General Mathematical Framework and Curation Strategy

Given an Industry 4.0 platform T (1), a set of N different generic sensor nodes $S_{i}$ are generating N independent data series $y_{i}\left[n\right]$. These data series suffer different malfunctions, and they are received by high-level software modules as a different set of N data series $x_{i}\left[n\right]$. These malfunctions are represented as a collection of L different functions $\lambda_{l}$ (2) transforming the original series generated by the sensor nodes $y_{i}\left[n\right]$ into de the received time series $x_{i}\left[n\right]$.

(1) $$T=\left\{S_{i},\;\;i=1,\ldots ,N\right\}$$

(2) $$x_{i}\left[n\right]=\lambda_{1}{\;}^{{}^{\circ}}\ldots .{\;}^{{}^{\circ}}\lambda_{l}{\;}^{{}^{\circ}}\ldots {\;}^{{}^{\circ}}\lambda_{L}\left(y_{i}\left[n\right]\right)$$

Although other sensing patterns could be applied in industrial scenarios, samples are periodically generated and sent to the high-level software modules for real-time monitoring and decision making. Samples in the $S_{i}$ sensor node are produced each $T_{i}$ seconds (3).

(3) $$x_{i}\left[n\right]=x_{i}\left(nT_{i}\right)\;\;\;\;\;n\;\in \;\mathbb{N}$$

Statically, each data series $x_{i}\left[n\right]$ is a realization of a stochastic process $\phi_{i}$ (4), where Ω is the universe of possible values $\omega_{k}$ generated by the sensor node $S_{i}$. This universe is a subset of the field of real numbers ℝ (5). This universe is discrete and strictly depends on the hardware capabilities of the sensor node, and it is analyzed and reported by manufacturers.

(4) $$\phi_{i}\left(n,\omega_{k}\;\right)=x_{i}\left[n\right]\;\;\;\;\omega_{k}\in \Omega \;$$

(5) $$\Omega =\left\{\omega_{k}\;\;\;k=1,\ldots ,K\right\}\;\subset \;\mathbb{R}$$

For each different time instant $n_{0}$, the stochastic process $\phi_{i}$ transforms into a different random variable $X_{i}\left[\omega \right]$ (6) with some specific statistical properties.

(6) $$\phi_{i}\left(n_{0},\omega \;\right)=X_{i}\left[\omega \right]$$

However, in Industry 4.0 scenarios, physical variables evolve much slower than the sampling period $T_{i}$; i.e., the superior frequency of physical signals $f_{max}\;$is much lower than the sampling frequency $f_{s}^{i}$ (7).

(7) $$f_{max}\ll \frac{1}{T_{i}}=f_{s}^{i}$$

In this context, for any time instant $n_{0},$ it is possible to define an open time interval $B_{s}$ around $n_{0}\;$with radix ε (8), where the random variables show equivalent statistical properties for all time instants. Thus, we are assuming the stochastic process $\phi_{i}\;$is locally first-order stationary in $B_{s}$ (9).

(8) $$B_{s}=\left\{n\;\in \;\mathbb{N}\;:d\left(n,n_{0}\right)<\varepsilon \right\}\;being\;d\left(n,n_{0}\right)=\sqrt{{\left(n-n_{0}\right)}^{2}}\;the\;Euclidean\;distance\;in\;\mathbb{R}$$

(9) $$\phi_{i}\left(n,\omega \;\right)=\phi_{i}\left(n+n_{c},\omega \;\right)\;\;\;\forall \;n,\;n+n_{c}\;\in B_{s}$$

Given a data series $x_{i}\left[n\right]$, if data in the time interval $\left[n_{1},\;n_{2}\right]$ should be curated, an expanded time interval $\left[n_{1}^{e},\;n_{2}^{e}\right]$ (10) must be considered, so it contains the original interval $\left[n_{1},\;n_{2}\right]$ where data must be curated, but it is included in the open time interval $B_{s}$ (the stochastic process must be stationary in the interval).

(10) $$\left[n_{1},\;n_{2}\right]\subset \left[n_{1}^{e},\;n_{2}^{e}\right]\subset \;B_{s}$$

This time interval $\left[n_{1},\;n_{2}\right]$ may refer to a past time period (11) (so we are performing an offline data curation), but we can also perform a real-time data curation if the current time instant $n_{0}$ belongs to the time interval $\left[n_{1},\;n_{2}\right]$ under study (12). If operating in real time, the proposed curation solution is employed as a prediction mechanism for calculating future samples in advance. If offline data curation is performed, the algorithm may be run as fast as possible, while in real-time data curation, the process is synchronized with the sampling period $T_{i}$, so one sample is curated at each time instant, although as many samples as desired may be predicted with each new sampling period $T_{i}$.

(11) $$n_{0}\;\notin \left[n_{1},\;n_{2}\right]\;:\;\;\;n_{0}<n_{1}$$

(12) $$n_{0}\;\in \left[n_{1},\;n_{2}\right]\;:\;\;n_{1}\leq n_{0}<n_{2}$$

The problem of data curation is to find a new time series $x_{i}^{*}\left[n\right]$ in the interval $\left[n_{1},\;n_{2}\right]$, so it represents in a more precise way (compared to the original time series $x_{i}\left[n\right]$) the real situation of the Industry 4.0 system, represented by time series $y_{i}\left[n\right]$. As many random effects impact this study, a probabilistic approach is the most adequate, so this condition transforms in a comparison between two different probabilities, $p_{i}^{*}$ and $p_{i}$ (13). Hereinafter, $P\left(\cdot \right)$ is the probability function, calculating the probability of a predicate to be true.

(13) $$p_{i}^{*}>p_{i}\;p_{i}^{*}=P\left(x_{i}^{*}\left[n\right]=y_{i}\left[n\right]\;\;\;\forall n\;\in \;\left[n_{1},\;n_{2}\right]\right)=P\left(x_{i}^{*}\left[n\right]\right)\;p_{i}=P\left(x_{i}\left[n\right]=y_{i}\left[n\right]\;\;\;\forall n\;\in \;\left[n_{1},\;n_{2}\right]\right)=P\left(x_{i}\left[n\right]\right)$$

**Figure 1:**
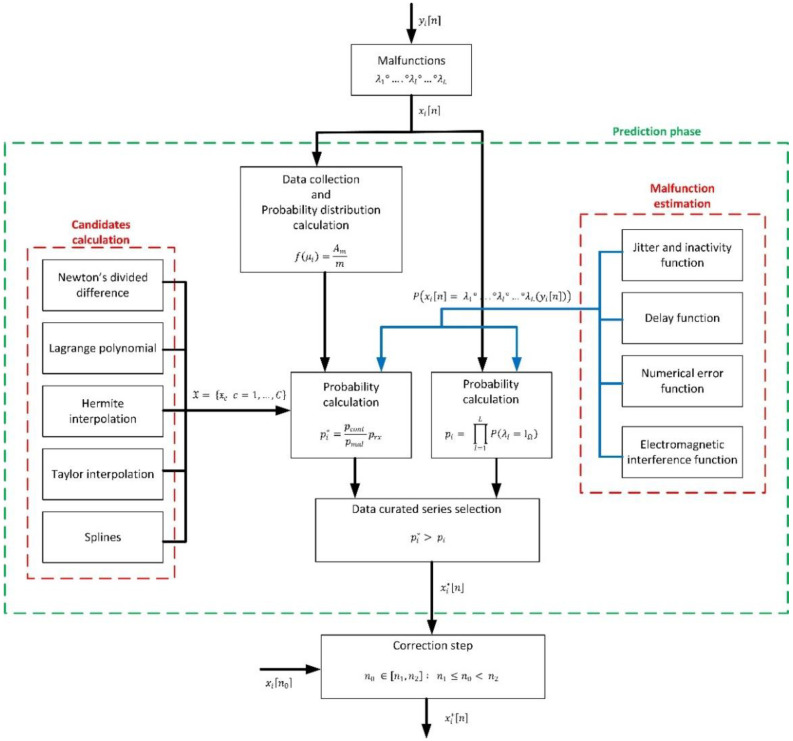
Global overview of the proposed prediction-correction algorithm.

Figure 1 shows the proposed algorithm to find that time series $x_{i}^{*}\left[n\right]$, fulfilling the previous conditions (13), if it exists.

As can be seen (in the initial prediction phase), first, a set of C suitable candidates X to be that curated time series $x_{i}^{*}\left[n\right]$ are calculated (14).

(14) $$X=\left\{{𝔵}_{c}\;\;c=1,\ldots ,C\right\}$$

To calculate those candidates, different techniques are employed, based on interpolation mechanisms. In particular, five different techniques are considered: Newton’s divided differences, Hermite interpolation, splines, Taylor interpolation and Lagrange polynomial. The purpose of this approach is to guarantee the curated time series $x_{i}^{*}\left[n\right]$ is continuous and coherent with samples outside the curation interval $\left[n_{1},\;n_{2}\right]$. Using the previously curated data in the expanded interval $\left[n_{1}^{e},\;n_{2}^{e}\right]$, a collection of possible curated time series $x_{i}^{*}\left[n\right]$ are calculated, considering all samples define a continuous function. For each one of these candidates ${𝔵}_{c}$, then, it is applied the statistical theory (Bayes’ theorem) to obtain probability $p_{i}^{*}$. If, for any candidate, the curation condition (13) is met, that candidate ${𝔵}_{c}$ is selected as the curated time series $x_{i}^{*}\left[n\right]$. On the contrary, and depending on how different probabilities $p_{i}^{*}$ and $p_{i}$ are, the time series may remain as is, or the curate data series may be obtained as a combination of the most probable candidates and the original data flow $x_{i}\left[n\right]$.

If a real-time data curation is performed, new information about the curation interval $\left[n_{1},\;n_{2}\right]$ is received at each sampling period $T_{i}$. In that case, a correction phase is carried out. In this phase, the new sample is compared to the predicted one, and (depending on how different they are) different actions are taken to correct the curated time series initially calculated.

To solve this problem, both probabilities $p_{i}^{*}$ and $p_{i}$ must be obtained.

Probability $p_{i}$ represents the fact that the received data $x_{i}\left[n\right]$ are exactly those data generated by the sensor nodes $y_{i}\left[n\right]$. In other words, no malfunction (of any kind) has occurred in the interval $\left[n_{1},\;n_{2}\right]$. In our model, that means functions $\lambda_{l}$ are the identity function all of them (15). As all the malfunctions are physically independent, they are also statistically independent, and the joint probability may be rewritten as a product of unidimensional probabilities (16). Section 3.3 analyzes how to evaluate those probabilities for each one of the considered malfunctions.

(15) $$p_{i}=P\left(x_{i}\left[n\right]=\lambda_{1}{\;}^{{}^{\circ}}\ldots .{\;}^{{}^{\circ}}\lambda_{l}{\;}^{{}^{\circ}}\ldots {\;}^{{}^{\circ}}\lambda_{L}\left(y_{i}\left[n\right]\right)=y_{i}\left[n\right]\right)=P\left(\lambda_{l}=I_{\Omega }\;\;\forall \;l=1,\ldots ,L\right)$$

(16) $$p_{i}=\overset{L}{\underset{l=1}{\prod }}P\left(x_{i}\left[n\right]=\lambda_{l}\left(y_{i}\left[n\right]\right)=y_{i}\left[n\right]\right)=\overset{L}{\underset{l=1}{\prod }}P\left(\lambda_{l}=I_{\Omega }\right)$$

On the other hand, probability $p_{i}^{*}$ is more complicated to calculate, and the Bayes’ theorem is employed (17).

(17) $$p_{i}^{*}=\frac{P\left(x_{i}^{*}\left[n\right]\;|\;x_{i}\left[n\right]\;\forall \;n\;\in \left[n_{1}^{e},\;n_{2}^{e}\right]\right)}{P\left(x_{i}\left[n\right]\;\forall \;n\;\in \left[n_{1}^{e},\;n_{2}^{e}\right]\;|\;x_{i}^{*}\left[n\right]\;\right)}P\left(x_{i}\left[n\right]\;\forall \;n\;\in \left[n_{1}^{e},\;n_{2}^{e}\right]\;\right)=\frac{p_{cont}}{p_{mal}}p_{rx}$$

To apply this theorem, three different probabilities, $p_{cont}$, $p_{mal}\;$and $p_{rx}$, must be obtained. Probability $p_{mal}$ is the probability of functions $\lambda_{l}$ (representing the malfunctions) to transform data series $x_{i}^{*}\left[n\right]$ into series $x_{i}\left[n\right]$ in the interval $\left[n_{1},\;n_{2}\right]$. As said before, this probability may be written as a product of L different unidimensional probabilities (18). Section 3.3 analyzes how to evaluate those probabilities for each one of the considered malfunctions.

(18) $$p_{mal}=P\left(\lambda_{l}\left(x_{i}^{*}\left[n\right]\;\right)=x_{i}\left[n\right]\;\;\forall \;l=1,\ldots ,L\;\;\;n\in \left[n_{1},\;n_{2}\right]\right)=\overset{L}{\underset{l=1}{\prod }}P\left(\lambda_{l}\left(x_{i}^{*}\left[n\right]\;\right)=x_{i}\left[n\right]\;\right)$$

Probability $p_{rx}$ is the probability of receiving the sequence $x_{i}\left[n\right]$ in the interval $\left[n_{1}^{e},\;n_{2}^{e}\right]$. That variable may be easily calculated using the probability function of the random variable (and stochastic process) $\phi_{i}\left(n,\omega \;\right)$ in the time interval $B_{s}\;$(19). In this case, once again, we are considering samples are independent events, so the join probability may be rewritten as a product.

(19) $$p_{rx}=\phi_{i}\left(n,x_{i}\left[n\right]\;\;\forall n\in \;\left[n_{1}^{e},\;n_{2}^{e}\right]\right)=\underset{\forall n\in \;\left[n_{1}^{e},\;n_{2}^{e}\right]}{\prod }\phi_{i}\left(n,x_{i}\left[n\right]\;\right)$$

Finally, probability $p_{cont}$ is the probability of the Industry 4.0 system’s evolution to follow a continuous and coherent flow. In this case, we are evaluating how probable is the data series $x_{i}^{*}\left[n\right]$ to show certain values in the interval $\left[n_{1},\;n_{2}\right]$, considering the other data received in the expanded time interval $\left[n_{1}^{e},\;n_{2}^{e}\right]$. As the stochastic process is stationary in the interval $B_{s}$, the probability distribution $g_{i}^{0}$ the interval $\left[n_{1},\;n_{2}\right]$ and distribution $g_{i}^{e}$ in the expanded time interval $\left[n_{1}^{e},\;n_{2}^{e}\right]$ must be identical. As both distributions become different, the probability of series $x_{i}^{*}\left[n\right]$ to be the best candidate for the curated series is reduced.

To calculate how different these two distributions are, we are employing the traditional function scalar product and the Lebesgue integral (20). However, in this case, as the universe under study is discrete, the Lebesgue integral may be approximated by a common sum. Thus, and considering the distance function induced by the function scalar product, we can calculate the distance $d_{g}$ between distributions $g_{i}^{0}$ and $g_{i}^{e}$ (21). Finally, to calculate the probability $p_{cont}$, we must apply a function transforming values in the interval $\left[0,\infty \right)$ in the interval $\left[0,\;1\right]$ (22).

(20) $$\langle g_{i}^{0}\;,\;g_{i}^{e}\rangle =\int_{\Omega }^{}g_{i}^{0}\;\cdot \;g_{i}^{e}\;\;d\omega \;\approx \;\underset{\Omega }{\sum }g_{i}^{0}\;\cdot \;g_{i}^{e}$$

(21) $$d_{g}=d\left(g_{i}^{0}\;,\;g_{i}^{e}\right)=\sqrt{g_{i}^{0}\;,-g_{i}^{e},\;g_{i}^{0}\;,-g_{i}^{e}}=\sqrt{\underset{\Omega }{\sum }{\left(g_{i}^{0}-g_{i}^{e}\right)}^{2}}$$

(22) $$p_{cont}=1-e^{-d_{g}}$$

Then, to enable the calculation of probabilities $p_{cont}$ and $p_{cont}$, we have to model the probability distribution of the stochastic process $\phi_{i}$ within the interval $B_{s}$.

We are now defining the operator $ℭ\left(\cdot ,\cdot \right)$ within the universe Ω (23). Basically, this operator indicates the number of elements in the universe that are between two provided values; that is, $card\left\{\cdot \right\}$ the standard cardinality operator. This operator is coherent as Ω is a subset of the field of the real number where a strict order relation is defined. This operator is a positive operator as the target set is the set of natural numbers $\mathbb{N}\;\cup \;\left\{0\right\}$.

(23) $$ℭ\;:\;\Omega \;\times \;\Omega \;\to \;\mathbb{N}\;\cup \;\left\{0\right\}\;ℭ\left(\omega_{1},\omega_{2}\right)=card\left\{\omega_{k}\;\;:\;\;\omega_{1}\leq \omega_{k}<\omega_{2}\right\}$$

Now, we are assuming the stochastic process $\phi_{i}$ is also locally ergodic in $B_{s}$. Thus, and according to the Birkhoff ergodic theorem, the additions $A_{m}$ of the composite function $ℭ\;\circ \;\phi_{i}$ $\left(\mathrm{restricted}\;\mathrm{to}\;B_{s},\;\phi_{i}{\left.\right|}_{B_{s}}\right)$ converge “almost surely” to the statistical expected value of the composite function $ℭ\;\circ \;I_{\Omega }$ (24), where $I_{\Omega }$ is the identity function in the universe Ω.

(24) $$A_{m}=\overset{m}{\underset{r=0}{\sum }}\;\left(ℭ\;\circ \;{\left(\phi_{i}{\left.\right|}_{B_{s}}\right)}^{m}\right)\;\frac{A_{m}}{m}\;\to \;E\left[ℭ\;\circ \;I_{\Omega }\;\right]$$

Now, we are considering a partition $\Pi_{\Omega }$ of the universe Ω, composed of Δ different subsets $\pi_{i}$ (25). All subsets $\pi_{i}$ have the same measure ${𝓁}_{i}$, understood as the Lebesgue measure (26).

(25) $$\Pi_{\Omega }=\left\{\pi_{i}\;\;\;i=1,\ldots ,\Delta \right\}\;\Pi_{\Omega }=\overset{\Delta }{\underset{i=1}{⋃}}\pi_{i}$$

(26) $${𝓁}_{i}=𝓁\left(\pi_{i}\right)=b_{i}-a_{i}\;being\;\pi_{i}=\left(a_{i},b_{i}\right)\subset \;B_{s}\;\subset \;\mathbb{R}$$

If we restrict the previous operator $ℭ\left(\cdot ,\cdot \right)$ to any subset $\pi_{i}$, $ℭ\;{\left.\right|}_{\pi_{i}}$ and evaluate this operator in the limits $\left(a_{i},b_{i}\right)$ of this set $\pi_{i}$, the statistical expected value of the composite function $ℭ\;{\left.\right|}_{\pi_{i}}\;\circ \;I_{\pi_{i}}$ is “almost surely” identical to the expression for the Laplace rule employed to calculate the probability of an event (27).

(27) $$\;\frac{A_{m}}{m}=\frac{1}{m}\overset{m}{\underset{r=0}{\sum }}\;\left(ℭ\;{\left.\right|}_{\pi_{i}}\;\circ \;{\left(\phi_{i}{\left.\right|}_{B_{s},\pi_{i}}\right)}^{m}\right)=\;=\frac{1}{m}\;card\left\{x_{i}\left[n\right]\;\;:\;\;n\in B_{s}\;\;⋀\;\;a_{i}\leq x_{i}\left[n\right]<b_{i}\right\}\;\to E\left[ℭ\;{\left.\right|}_{\pi_{i}}\;\circ \;I_{\pi_{i}}\;\right]$$

In this case, the event under study is the fact a sensor node generates a sample belonging to $\pi_{i}$ in the time interval $B_{s}$. In conclusion, we are studying the probability distribution of the stochastic process $\phi_{i}$ in the interval $B_{s}$.

We are now defining a function $f\left(\cdot \right)$ associating the mean point $\sigma_{i}$ of every subset $\pi_{i}$ with the additions $A_{m}$ (28). In other words, through the additions $A_{m}$, we are generating a discrete probability function $f\left(\cdot \right)$, which “almost surely” converges to the actual probability distribution of the stochastic process $\phi_{i}$ in the interval $B_{s}$ and the points $\sigma_{i}$ (29).

(28) $$f\left(\sigma_{i}\right)=f\left(\frac{{𝓁}_{i}}{2}\right)=\frac{A_{m}}{m}=\frac{1}{m}\;card\left\{x_{i}\left[n\right]\;\;:\;\;n\in B_{s}\;\;⋀\;\;a_{i}\leq x_{i}\left[n\right]<b_{i}\right\}$$

(29) $$f\left(\sigma_{i}\right)\;\to \;\phi_{i}{\left.\right|}_{B_{s},\pi_{i}}\left(n_{0},\sigma_{i}\right)$$

To calculate the probability distributions $g_{i}^{0}$ and $g_{i}^{e}$, the same process as described before may be employed, but considering the proper time interval and a new universe Σ composed by points $\sigma_{i}$ (30). Probabilty $p_{rx}$ can be directly obtained using function $f\left(\sigma_{i}\right)$.

(30) $$\Sigma =\left\{\sigma_{i}\;\;\;i=1,\ldots ,\Delta \right\}$$

### 3.2. Candidates to Curated Time Series: Calculation

The first step to improve the quality of the time series $x_{i}\left[n\right]$ produced by sensor nodes $S_{i}$ in Industry 4.0 systems is to find the candidate series X to be the curated flow we are looking for. Initially, any series ${𝔵}_{c}\;$could be a candidate, and probability $p_{i}^{*}$ will be the indicator to select the final curated series $x_{i}^{*}\left[n\right]$. However, this approach is almost impossible to implement in practice, as the universe of time series in the curation interval $\left[n_{1},\;n_{2}\right]$ is infinite. Moreover, as this is not a free mathematical problem, some physical restrictions inherit from the Industry 4.0 system we are modeling must be considered.

First, sensor nodes have an operational range $\left[x_{min},\;x_{max}\right]$, which introduces a hard restriction: no candidate ${𝔵}_{c}\;$with samples in the exterior of the interval $\left[x_{min},\;x_{max}\right]$ is in the final curated series $x_{i}^{*}\left[n\right]$ (31).

(31) $${𝔵}_{c}\;\left[n\right]\notin \;\left[x_{min},\;x_{max}\right]\;\;n\;\in \left[n_{1}^{e},\;n_{2}^{e}\right]\;\;\;\Rightarrow \;{𝔵}_{c}\;\left[n\right]\neq \;x_{i}^{*}\left[n\right]\;\;\;\;n\;\in \left[n_{1}^{e},\;n_{2}^{e}\right]$$

Second, Industry 4.0 systems monitor physical processes, which are continuous and smooth (as natural variables), so no gaps or abrupt changes may appear in the curated time series. In this context, curated series $x_{i}^{*}\left[n\right]$ in the interval $\left[n_{1},\;n_{2}\right]$ must be continuous and coherent with time series in the surrounds of this interval, i.e., in the expanded time interval $\left[n_{1}^{e},\;n_{2}^{e}\right]$. In that way, analytic function describing the evolution of the Industry 4.0 system in the curation interval $\left[n_{1},\;n_{2}\right]$ must also be able to describe the system evolution in the expanded interval $\left[n_{1}^{e},\;n_{2}^{e}\right]$. To apply this restriction, the best way to calculate the candidates ${𝔵}_{c}$ is using interpolation techniques.

Different interpolation techniques may generate different candidates ${𝔵}_{c}$, so in this work, we are considering the most powerful, popular and well-behaved interpolation solutions: Newton’s divided differences, Hermite interpolation, splines, Taylor interpolation and Lagrange polynomial.

These techniques are evaluated using the E points $x_{ext}^{i}\left[n\right]$ which belong to the expanded interval $\left[n_{1}^{e},\;n_{2}^{e}\right],$ but they are not included in the curation interval $\left[n_{1},\;n_{2}\right]$ (as data in the curation interval may be wrong and introduce false information in our algorithm) (32).

(32) $$x_{ext}^{i}\left[n\right]=\left\{x_{ext}^{i}\left[n_{j}^{ext}\right]\;\;\;j=1,\ldots ,E\right\}=x_{i}\left[n\right]\;:\;\;\;n\in \;\left\{\left[n_{1}^{e},\;n_{2}^{e}\right]\;\cap \;\left[n_{1},\;n_{2}\right]\right\}=\;=\left\{n_{j}^{ext}\;j=1,\ldots ,E\right\}$$

Candidate ${𝔵}_{1}\;\left[n\right]$ is obtained using the Newton’s divided differences technique. In this case, as the independent variable is the discrete time n, traditional expressions for Newton’s interpolation are slightly modified. Specifically, given the E points in $x_{ext}^{i}\left[n\right]$, candidate ${𝔵}_{1}\;\left[n\right]$ is a polynomial with order E−1 (33). Coefficients (named as divided differences) may be easily calculated using simple mathematical operations, which reduces the computational time, enabling a real-time operation (34).

(33) $${𝔵}_{1}\;\left[n\right]=\delta_{0}+\overset{E}{\underset{z=1}{\sum }}\delta_{z}\cdot \overset{z}{\underset{r=1}{\prod }}\left(n-n_{r}^{ext}\right)$$

(34) $$\delta_{0}=x_{ext}^{i}\left[n_{1}^{ext}\right]\;\;\;\;\delta_{1}=\frac{x_{ext}^{i}\left[n_{2}^{ext}\right]-\delta_{0}}{n_{2}^{ext}-n_{1}^{ext}}\;\delta_{2}=\frac{\frac{x_{ext}^{i}\left[n_{3}^{ext}\right]-\delta_{0}}{n_{3}^{ext}-n_{2}^{ext}}-\delta_{1}}{n_{3}^{ext}-n_{1}^{ext}}\;\delta_{3}=\frac{\frac{\frac{x_{ext}^{i}\left[n_{3}^{ext}\right]-\delta_{0}}{n_{3}^{ext}-n_{2}^{ext}}-\delta_{1}}{n_{3}^{ext}-n_{1}^{ext}}-\delta_{2}}{n_{4}^{ext}-n_{1}^{ext}}\;\ldots$$

Candidate ${𝔵}_{2}\;\left[n\right]$ is calculated through the Lagrange polynomial interpolation algorithm. In this case, the candidate is just a linear combination of data in sequence $x_{ext}^{i}\left[n\right]$ (35). Besides, in this case, the order β of the interpolation polynomial may be selected (if it is lower than E, number of points in $x_{ext}^{i}\left[n\right]$). In general, polynomial with orders above six are not suitable (because they present unnatural fluctuations), but this parameter is free to be selected according to the Industry 4.0 system under study.

(35) $${𝔵}_{2}\;\left[n\right]=\overset{\beta }{\underset{z=1}{\sum }}x_{ext}^{i}\left[n_{z}^{ext}\right]\overset{\beta }{\underset{r=1\;\;r\neq z}{\prod }}\frac{n-n_{r}^{ext}}{n_{z}^{ext}-n_{r}^{ext}}\;\;\;\;\beta <E$$

The third candidate ${𝔵}_{3}\;\left[n\right]$ is obtained using the Hermite interpolation theory. In this case, besides the sequence $x_{ext}^{i}\left[n\right]$, it is also necessary to know the value of the first order derivative $\dot{x_{ext}^{i}}\left[n\right]$ in the points $n_{j}^{ext}$. When managing discrete-time sequences, this may be easily calculated using first-order finite differences. In general, we are using a central difference (36), as it presents a much lower error. However, if either time point $n_{j-1}^{ext}$ or time point $n_{j+1}^{ext}$ do not exit, we can employ the forward difference (37) or the backward difference (38) respectively (and although a higher numerical error is introduced).

(36) $$\dot{x_{ext}^{i}}\left[n_{j}^{ext}\right]=\frac{x_{ext}^{i}\left[n_{j+1}^{ext}\right]-x_{ext}^{i}\left[n_{j-1}^{ext}\right]}{n_{j+1}^{ext}-n_{j-1}^{ext}}$$

(37) $$\dot{x_{ext}^{i}}\left[n_{j}^{ext}\right]=\frac{x_{ext}^{i}\left[n_{j+1}^{ext}\right]-x_{ext}^{i}\left[n_{j}^{ext}\right]}{n_{j+1}^{ext}-n_{j}^{ext}}\;\;\;j<1$$

(38) $$\dot{x_{ext}^{i}}\left[n_{j}^{ext}\right]=\frac{x_{ext}^{i}\left[n_{j}^{ext}\right]-x_{ext}^{i}\left[n_{i-1}^{ext}\right]}{n_{j}^{ext}-n_{j-1}^{ext}}\;\;\;j>E$$

If both time instants $n_{j-1}^{ext}$ and $n_{j+1}^{ext}$ do not exist, the first-order derivative cannot be calculated for instant $n_{j}^{ext}$. In that case, that point $n_{j}^{ext}$ is not considered to calculate the candidate sequence ${𝔵}_{3}\;\left[n\right]$.

Given the Lagrange polynomial $L_{r}\;\left[n\right]$ (39), and its first order derivative $\dot{L_{r}}\;\left[n\right]$, the Hermite interpolated sequence may be calculated through an osculating polynomial (40). This approach generates high-quality candidates, which may integrate large amount of points with a reduced computational cost.

(39) $$L_{r}\;\left[n\right]=\overset{E}{\underset{z=1\;\;r\neq z}{\prod }}\frac{n-n_{z}^{ext}}{n_{r}^{ext}-n_{z}^{ext}}$$

(40) $${𝔵}_{3}\;\left[n\right]=\overset{E}{\underset{r=1}{\sum }}x_{ext}^{i}\left[n_{r}^{ext}\right]\cdot H_{r}\left[n\right]+\overset{E}{\underset{r=1}{\sum }}\;\dot{x_{ext}^{i}}\left[n_{r}^{ext}\right]\cdot \hat{H_{r}}\left[n\right]\;H_{r}\left[n\right]=\left(1-2\left(n-n_{r}^{ext}\right)\dot{L_{r}}\;\left[n\right]\right)\cdot L_{r}^{2}\;\left[n\right]\;\hat{H_{r}}\left[n\right]=\left(n-n_{r}^{ext}\right)\cdot L_{r}^{2}\;\left[n\right]$$

Candidate ${𝔵}_{4}\;\left[n\right]$ is based on Taylor’s interpolation. Formally, this approach only requires one sample at the time instant $n_{taylor}^{ext}$, so it is a very good candidate for the initial moments of the Industry 4.0 system operation, when collected data are very limited. In practice, however, this method requires the use of different successive r-th derivatives $\overset{\left(z\right)}{\overset{︷}{x_{ext}{}^{i}}}\left[n\right]$. They can be easily obtained using the central, forward or backward differences we already described (36)–(38), but this needs some additional samples. In this approach, the order β of the interpolation polynomial can be also selected. Therefore, in general, for a given order β, this method needs between β+1 and β+2 samples. As said before, polynomial with orders above six show some unnatural variations. On the other hand, for very low values of β, the numerical error is also high. A balance between both factors must be reached.

In this context, candidate ${𝔵}_{4}\;\left[n\right]$ may be easily obtained (41).

(41) $${𝔵}_{4}\;\left[n\right]=\overset{\beta }{\underset{z=1}{\sum }}\frac{\overset{\left(z\right)}{\overset{︷}{x_{ext}{}^{i}}}\left[n_{taylor}^{ext}\right]}{z!}{\left(n-n_{taylor}^{ext}\right)}^{z}\;\;\;\;\beta <E$$

Finally, candidate ${𝔵}_{5}\;\left[n\right]$ is obtained through splines. Using the splines technique, candidate is just a segmented polynomial (42).

(42) $${𝔵}_{5}\;\left[n\right]=\left\{\begin{array}{c} {𝔵}_{5}^{1}\;\left[n\right]\;\;\;\;n\in \left[n_{1}^{ext},n_{2}^{ext}\right)\\ \ldots \\ {𝔵}_{5}^{j}\;\left[n\right]\;\;\;\;n\in \left[n_{j}^{ext},n_{j+1}^{ext}\right)\\ \ldots \\ {𝔵}_{5}^{E-1}\;\left[n\right]\;\;\;\;n\in \left[n_{E-1}^{ext},n_{E}^{ext}\right) \end{array}\right.$$

This polynomial may have different orders (from one to three), but it is well-known that cubic splines is the solution generating the best candidates [53] (they are smooth, contrary to linear splines, and they adapt to a larger range of system behaviors). For each pair of time instants $n_{j}^{ext}$, $n_{j+1}^{ext}$ a new cubic polynomial ${𝔵}_{4}^{j}\;\left[n\right]$ is defined (43). In this polynomial ${𝔵}_{5}^{j}\;\left[n\right]$, variables $\mu_{j}$ are unknown and are calculated through the continuity and smoothness conditions (44). In this proposal, we are using natural splines, so for any point $n_{j}^{ext}$ where it is impossible to calculate the first-order derivative $\dot{{𝔵}_{5}^{j}}\;\left[n\right]$ or the second-order derivative $\ddot{{𝔵}_{5}^{j}}\;\left[n\right]$, these values are considered null (zero).

(43) $${𝔵}_{5}^{j}\;\left[n\right]=\frac{\mu_{j}}{6\left(n_{j+1}^{ext}-n_{j}^{ext}\right)}{\left(n_{j+1}^{ext}-n\right)}^{3}+\frac{\mu_{j+1}}{6\left(n_{j+1}^{ext}-n_{j}^{ext}\right)}{\left(n-n_{j}^{ext}\right)}^{3}\;+\left(\frac{x_{ext}^{i}\left[n_{j+1}^{ext}\right]}{n_{j+1}^{ext}-n_{j}^{ext}}+\frac{\mu_{j+1}\left(n_{j+1}^{ext}-n_{j}^{ext}\right)}{6}\right)\cdot \left(n-n_{j}^{ext}\right)\;+\left(\frac{x_{ext}^{i}\left[n_{j}^{ext}\right]}{n_{j+1}^{ext}-n_{j}^{ext}}-\frac{\mu_{j}\left(n_{j+1}^{ext}-n_{j}^{ext}\right)}{6}\right)\cdot \left(n_{j+1}^{ext}-n\right)$$

(44) $${𝔵}_{5}^{j}\;\left[n_{j}^{ext}\right]={𝔵}_{5}^{j+1}\;\left[n_{j}^{ext}\right]\;\;\;\;j=1,\ldots ,E-1\;\dot{{𝔵}_{5}^{j}}\;\left[n_{j}^{ext}\right]=\dot{{𝔵}_{5}^{j+1}}\;\left[n_{j}^{ext}\right]\;\;\;\;j=1,\ldots ,E-1\;\ddot{{𝔵}_{5}^{j}}\;\left[n_{j}^{ext}\right]=\ddot{{𝔵}_{5}^{j+1}}\;\left[n_{j}^{ext}\right]\;\;\;\;j=1,\ldots ,E-1$$

This final candidate is more computationally costly to calculate, as we are introducing a system of linear equations that must be solved to obtain the final expression for the candidate. However, this method creates high-quality, curated data series (with a reduced error), and (currently) linear equations are easily solved using numerical methods (especially in strong cloud infrastructures or Industry 4.0 software platforms).

### 3.3. Malfunction Modeling

The calculation of probabilities $p_{i}$ and $p_{mal}\;$is directly associated to functions $\lambda_{l}$, which model the data malfunctions in the Industry 4.0 platform. In this proposal, four different malfunctions are addressed: jitter (including inactivity periods in hardware nodes), communication delays, numerical errors in microprocessors and electromagnetic interferences (including data losses and transmissions errors). As said before, to calculate $p_{i}$, we must consider the probability of all these functions $\lambda_{l}$ to be the identity function $I_{\Omega }$ (null effect), while probability $p_{mal}$ is obtained considering (for each candidate) the situation when $\lambda_{l}\left({𝔵}_{c}\;\left[n\right]\;\right)=x_{i}\left[n\right]$.

Jitter $J\left(\xi \right)$ is probably the most harmful malfunction among all the ones considered in this proposal. Jitter is the maximum fluctuation in the communication delays, transmission periods or clock synchronization that causes samples $x_{i}\left[n\right]$ to be randomly ordered compared to the original ones in $y_{i}\left[n\right]$ (45). Jitter is represented by a function $\tau_{jitter}\left[k\right]$ taking values in the interval $\left[0,\xi_{k}\;\right]$, where $\xi_{k}$ is a realization (different for each value of k) of a random variable $J\left(\xi \right)$ taking values in a continuous universe: the field of positive real numbers (46). Because of jitter, no samples may be received for long periods, while in other moments, large amounts of samples may be received and interfere.

(45) $$x_{i}\left[n\right]=\lambda_{1}\left(y_{i}\left[n\right]\;\right)=\underset{\forall \;k\;\;:\;\;n=k-\tau_{jitter}\left[k\right]\;}{\sum }y_{i}\left[k-\tau_{jitter}\left[k\right]\;\right]$$

(46) $$\tau_{jitter}\left[k\right]\;\in \;\left[0,\xi_{k}\;\right]\;P\left(\xi_{k}\right)=J\left(\xi_{k}\right)\;\;\;\xi_{k}\;\in \mathbb{R}\;⋃\;\left\{0\right\}$$

Total jitter $J\left(\xi \right)$ in Industry 4.0 systems is, actually, the composition of two different and independent sources: random jitter $J_{random}\left(\xi \right)$ and deterministic jitter $J_{deter}\left(\xi \right)$ (47). Operator * represents the convolution.

Deterministic jitter is caused by three different additive effects (well-known and modeled through deterministic expressions): fluctuations in the clock periods and edges, variations in the data packet length and sleep periods in the sensor nodes or the communication channels [54]. Although these three effects are deterministic, they are also dependent on random variables, such as the clock-duty cycle and frequency, the packet length, and the duration of the blocking, respectively. In conclusion, deterministic jitter $J_{deter}\left(\xi \right)$ is a Gaussian distribution, according to the central limit theorem (48) with mean value $m_{det}$ and standard deviation $s_{det}$.

(47) $$J\left(\xi \right)=J_{deter}\left(\xi \right)*J_{random}\left(\xi \right)$$

(48) $$J_{deter}\left(\xi \right)=\frac{1}{s_{det}\sqrt{2\pi }}\;e^{-\frac{{\left(\xi -m_{det}\right)}^{2}}{2s_{det}^{2}}}$$

On the other hand, many other random and uncontrolled effects, such as thermal oscillations, flicker or shot noise, may result in levels of jitter that cannot be predicted or calculated in any way. This is known as random jitter $J_{random}\left(\xi \right)$ and, according to the central limit theorem, is also a Gaussian distribution (49) with mean value $m_{ran}$ and standard deviation $s_{ran}$.

(49) $$J_{random}\left(\xi \right)=\frac{1}{s_{ran}\sqrt{2\pi }}\;e^{-\frac{{\left(\xi -m_{ran}\right)}^{2}}{2s_{ran}^{2}}}$$

The convolution of these two Gaussian distributions is a third Gaussian distribution (50). The mean value m and the standard deviation s depend on the scenario and Industry 4.0 system, but most modern 5G solutions establish the mean value m around 1 millisecond and the standard deviation s is one magnitude order lower. These values are aligned with the expected performance for ultra-reliable low latency communications (URLLC) in 5G networks and scenarios [55].

(50) $$J\left(\xi \right)=\frac{1}{\sqrt{2\pi }\;\sqrt{s_{ran}^{2}+s_{\det}^{2}}}\;e^{-\frac{{\left(\xi -m_{ran}-m_{det}\right)}^{2}}{2\left(s_{ran}^{2}+s_{det}^{2}\right)}}=\frac{1}{s\sqrt{2\pi }}\;e^{-\frac{{\left(\xi -m\right)}^{2}}{2s^{2}}}$$

Communication delays $D\left(\xi \right)$ are not as harmful as jitter as they are a linear transformation (51), but they may cause delayed decisions and other similar problems. As all malfunctions, delays are a random effect, and they are described by a random variable taking values in a continuous universe: the field of positive real numbers (51).

In this case, we are decomposing the total delay $D\left(\xi \right)$ in three different contributions (52): delay in the output queue (sensor node) $D_{out}\left(\xi \right)$, transmission delay $D_{tran}\left(\xi \right)$, and delay in the input queue (software module) $D_{in}\left(\xi \right)$.

(51) $$x_{i}\left[n\right]=\lambda_{2}\left(y_{i}\left[n\right]\;\right)=\;y_{i}\left[n-\xi \right]\;P\left(\xi \right)=\;D\left(\xi \right)\;\;\;\xi \;\in \mathbb{R}\;⋃\;\left\{0\right\}$$

(52) $$D\left(\xi \right)=D_{out}\left(\xi \right)+D_{tran}\left(\xi \right)+D_{in}\left(\xi \right)$$

Both delays associated with queues are formally identical. The traffic theory allows obtaining the probability distribution for both components $D_{out}\left(\xi \right)$ and $D_{in}\left(\xi \right)$. In both cases, we are using a Poisson model M/M/1/P (in Kendall notation), where the sample generation rate ψ and the serving rate η follow a Poisson distribution, Ψ is the mean sample generation rate, Θ is the mean serving time and Γ is the time period taken as reference (typically one hour). P is the number of samples allowed in the system (53).

If we assume a FIFO (first in, first out) managing strategy for both queues in our model, we can define a Markov chain for describing the queues state. In that situation, the traffic theory and statistical laws define the queueing delay as an exponential distribution (54).

(53) $$\psi \left(z\right)=\frac{{\left(\Psi \cdot \;\Gamma \right)}^{z}}{z!}exp\left(-\Psi \cdot \;\Gamma \right)\;\eta \left(z\right)=\frac{{\left(\Theta \cdot \;\Gamma \right)}^{z}}{z!}exp\left(-\Theta \cdot \;\Gamma \right)$$

(54) $$D_{out}\left(\xi \right)∼D_{in}\left(\xi \right)={\left(\frac{\psi }{\eta }\right)}^{\eta \cdot \xi }\frac{1-\frac{\psi }{\eta }}{1-{\left(\frac{\psi }{\eta }\right)}^{P+1}}$$

Regarding the transmission delay $D_{tran}\left(\xi \right)$, several different random and unknown variables affect the final value: data packet length, channel capacity, physical configuration of the scenario, etc. Therefore, and considering the central limit theorem, the probability distribution of the transmission delay is a Gaussian function with mean value $m_{d}$ and standard deviation $s_{d}$ (55).

(55) $$D_{tran}\left(\xi \right)=\;\frac{1}{s_{d}\sqrt{2\pi }}\;e^{-\frac{{\left(\xi -m_{d}\right)}^{2}}{2s_{d}^{2}}}\;$$

Numerical errors in microprocessors are caused by the limited precision of hardware components. Basically, these errors are associated with two different modules: the analog-to-digital converter (ADC) and the arithmetic combinational modules. In the ADC, because of the quantification process, samples are irreversibly modified. Because of the arithmetic combinational modules, operations with large numbers may be truncated to the maximum number admissible in the microprocessor, $\rho_{max}$.

In any case, the numerical error $N\left(\xi \right)$ is an additive effect (56), composed of two different sub-effects: the ADC error $N_{ADC}\left(\xi \right)$ and the arithmetic error $N_{ari}\left(\xi \right)$ (57). In this case, random variable $N\left(\xi \right)$ takes values in the universe of real numbers (56).

(56) $$x_{i}\left[n\right]=\lambda_{3}\left(y_{i}\left[n\right]\;\right)=\;y_{i}\left[n\right]+\;\xi \;\;P\left(\xi \right)=\;N\left(\xi \right)\;\;\;\;\xi \;\in \mathbb{R}$$

(57) $$N\left(\xi \right)=N_{ari}\left(\xi \right)+N_{ADC}\left(\xi \right)$$

Regarding the quantification error in the ADC, we are assuming the sensor node is configured according to the manufacturer’s restrictions, and the analog signal $y_{i}\left(t\right)$ being digitalized is limited in amplitude to the operation rage $\left[-Y_{max}^{ADC},\;Y_{max}^{ADC}\right]$ of the ADC device. In this context, given an ADC device with u intervals with an amplitude of Σ units (58), the error will be restricted to the interval $\left[-\frac{\Sigma }{2},\frac{\Sigma }{2}\right]$ (the maximum error appears when the sample is exactly in the middle of an ADC interval) (59). All values within the proposed interval have the same probability, so the distribution is uniform (60).

(58) $$\Sigma =\frac{Y_{max}^{ADC}}{u}\;$$

(59) $$N_{ADC}\left(\xi \right)\;\;:\;\xi \;\in \;\left[-\frac{\Sigma }{2},\frac{\Sigma }{2}\right]$$

(60) $$N_{ADC}\left(\xi \right)=\frac{1}{\Sigma }\;\;\;\;\forall \;\xi$$

On the other hand, errors caused by the limited precision of arithmetic devices, $N_{ari}\left(\xi \right)$, may take any value as there is no superior limit. However, all these values do not have the same probability, as lower errors are much more probable than higher errors (input parameters are also limited and system designers usually also consider this problem in their code). In particular, we are proposing the arithmetic error follows an exponential distribution (61). If we assume the maximum number the microprocessor can represent is $\rho_{max}$, the worst situation to happen (where arithmetic error is highest) is the addition of two parameters with this maximum value $\rho_{max}$. The result, then, presents a maximum error of $\rho_{max}$ units. Thus, the mean value for our exponential distribution is $\rho_{max}$ (and the distribution gets totally defined).

(61) $$N_{ari}\left(\xi \right)=\frac{1}{\rho_{max}}\cdot e^{-\frac{\xi }{\rho_{max\;}}}$$

Finally, electromagnetic interferences are responsible for two main problems: sample losses and data corruption. At the data level, electromagnetic interferences show as a bit error rate (BER). If, because of BER, the number of corrupted bits in a sample $x_{i}\left[n\right]$ is above a limit $\gamma_{lim}$, the sample is corrupted and is rejected and not received. If the number of corrupted bits is below that limit $\gamma_{lim}$, the sample $x_{i}\left[n\right]$ is corrupted by an additive effect but is still received (62). Hereinafter, we are considering Λ as the length (in bits) of samples. This is a random variable with a uniform distribution in the interval $\left[0,\;\Lambda_{max}\right]$.

(62) $$x_{i}\left[n\right]=\lambda_{4}\left(y_{i}\left[n\right]\;\right)=\left\{\begin{array}{c} null\;\;\;\;\;\;\;\;\;\;\;\;\;\;\;if\;\;BER\cdot \Lambda >\gamma_{lim}\;\\ x_{i}\left[n\right]+\xi \;\;\;\;\;\;\;\;\;if\;\;BER\cdot \Lambda <\gamma_{lim} \end{array}\right.$$

(63) $$P\left(\Lambda \right)=\frac{1}{\Lambda_{max}}$$

The calculation of the additive distortion ξ is also based on the length of samples Λ (in bits). The error, then, must be included in the interval $\left[-\left(2^{\Lambda }-1\right),\;2^{\Lambda }-1\right]$, the maximum value that can be represented using Λ bits. Errors have the same probability in all bits, so the probability distribution of distortion values ξ is uniform.

(64) $$P\left(\xi \right)=\frac{1}{2^{\Lambda +1}}\;\;\xi \in \left[-\left(2^{\Lambda }-1\right),\;2^{\Lambda }-1\right]$$

Finally, we must calculate the value of BER using the level of electromagnetic interferences. Given the signal power per bit $E_{b}$ and the power of interferences $N_{0}$, the signal theory establishes the BER is obtained through the complementary error function $erfc\left(\cdot \right)$ (65).

(65) $$BER=\;\frac{1}{2}erfc\left(\sqrt{\frac{E_{b}}{N_{0}}}\right)=\frac{2}{\sqrt{\pi }}\int_{\sqrt{\frac{E_{b}}{N_{0}}}}^{\infty }e^{-t^{2}}dt$$

### 3.4. Final Data Generation and Correction Step

After calculating probabilities $p_{i}^{*}$ and $p_{i}$ and selecting the candidate ${𝔵}_{c}\;\left[n\right]$ associated to the higher probability $p_{i}^{*}$ among all five calculated candidates, different situations may occur. First, if offline data curation is being performed, no new information is expected after the performed calculations. Then, results are final. However, three possibilities are considered:

- If probability $p_{i}^{*}$ is clearly higher than $p_{i}$, candidate ${𝔵}_{c}\;\left[n\right]$ associated to this probability $p_{i}^{*}$ is selected as the curated data series $x_{i}^{*}\left[n\right]$ and initially received information $x_{i}\left[n\right]$ is deleted. In this proposal, we are considering a difference higher than 10% (66) as the limit to take this action.
  (66) $$\frac{p_{i}^{*}}{p_{i}}\;\geq 1.1$$
- On the contrary, if probability $p_{i}$ is clearly higher than probability $p_{i}^{*}$ (67), all candidates are rejected and initially received data flow $x_{i}\left[n\right]$ is accepted as the curated data series $x_{i}^{*}\left[n\right]$.
  (67) $$\frac{p_{i}}{p_{i}^{*}}\;\geq 1.1$$
- If neither probability $p_{i}^{*}$ nor $p_{i}$ is clearly higher than the other (68), the final curate data series $x_{i}^{*}\left[n\right]$ cannot be concluded to be the candidate ${𝔵}_{c}\;\left[n\right]$ or the originally received data $x_{i}\left[n\right]$. Thus, the curated data series $x_{i}^{*}\left[n\right]$ is obtained as the arithmetic average of both series (69).
  (68) $$0.9<\frac{p_{i}}{p_{i}^{*}}<1.1$$
  (69) $$x_{i}^{*}\left[n\right]=\frac{1}{2}\left(x_{i}\left[n\right]+{𝔵}_{c}\;\left[n\right]\right)\;\;\;\;\;n\in \;\left[n_{1},\;n_{2}\right]\;$$

Second, if real-time data curation is being performed, there will be a future time instant $n_{fut}$, belonging to the expanded interval $\left[n_{1}^{e},\;n_{2}^{e}\right]$, whose associated sample $x_{i}\left[n_{fut}\right]$ is not available at the time instant $n_{0}$ when the curated series is obtained, but it is received in the future. This new information may affect the previous calculation $x_{i}^{*old}\left[n\right]$, then a correction phase is considered to update the previous results.

First, the new sample $x_{i}\left[n_{fut}\right]$ is compared to the previously obtained curated sample $x_{i}^{*old}\left[n_{fut}\right]$. Then, if the difference is high enough (above 10%) (70), all the curation process is redone considering the new information.

(70) $$\frac{x_{i}\left[n_{fut}\right]}{x_{i}^{*old}\left[n_{fut}\right]}\;\geq 1.1\;\;\vee \;\frac{x_{i}\left[n_{fut}\right]}{x_{i}^{*old}\left[n_{fut}\right]}\leq 0.9$$

However, if the difference between samples $x_{i}\left[n_{fut}\right]$ and $x_{i}^{*old}\left[n_{fut}\right]$ is not relevant, to reduce the global computational cost of the proposed solution, the corrected curated data series $x_{i}^{*}\left[n_{fut}\right]$ is obtained as the arithmetic average of old curated sample $x_{i}^{*old}\left[n_{fut}\right]$ and the new information $x_{i}\left[n_{fut}\right]$ (71).

(71) $$x_{i}^{*}\left[n_{fut}\right]=\frac{1}{2}\left(x_{i}^{*old}\left[n_{fut}\right]+x_{i}\left[n_{fut}\right]\right)$$

Table 1 summarizes the most relevant symbols and variables considered in the proposed predictor-corrector scheme.

## 4. Experimental Validation and Results

To evaluate the performance and usability of the proposed technology, an experimental validation was designed and carried out. In this experimental phase, we analyzed the precision of the proposed prediction-correction method, but we also evaluated its behavior in terms of scalability and required computational time. This section describes the proposed experimental methods and the obtained results.

### 4.1. Experiments: Methods and Materials

Four different experiments were planned and developed. Three of them were based on simulation techniques, while the fourth one was supported by a real hardware infrastructure.

The first experiment evaluated the evolution of the computational cost of the proposed solution when deployed in an Industry 4.0 system. To do that, the number of sensor nodes N in the scenario was varied, and the total computational time required to curate all data series in real time was monitored. This experiment was focused on the solution’s cost and its scalability. Time measurements and results were captured and displayed as relative values normalized by the sampling period $T_{i}$. In that way, when processing time was above the sampling period $T_{i}$, we determined the proposed framework was congested (buffers will grow until the entire software module becomes unavailable). In this experiment, MATLAB 2019B software was employed to build a simulation scenario where we could change the number of nodes N in an easy way. The experiment was repeated for different values of the sampling period $T_{i}$.

The second experiment was also focused on analyzing the computational cost of the proposed solution and its scalability, but in this case when performing an offline data curation. In this case, the relative length of the expanded time interval $\left[n_{1}^{e},\;n_{2}^{e}\right]$, with respect to the curation interval $\left[n_{1},\;n_{2}\right]$, (72), was varied in different experiment’s realizations, and the total computational time required to curate all the samples in the curation interval was monitored.

(72) $$\frac{n_{2}^{e}-n_{1}^{e}}{n_{2}-n_{1}}$$

As in the first experiment, to reduce the external validity threats to this experiment and make results more general, computational time was expressed as a relative number respect to the sampling period $T_{i}$. This experiment was also based on simulation techniques using the MATLAB 2019B software, where an Industry 4.0 system was built and run. All sensors were generating data every 30 s. The experiment was repeated for different numbers of sensor nodes in the Industry 4.0 system, N.

The third experiment aimed to evaluate the precision and curation success of the proposed predictive-correction algorithm. In this case, the experiment was also based on simulation techniques using the MATLAB 2019B software. Measures about the proposal’s precision were obtained as the mean square error, MSE, (73) for the entire curated data flow and all the sensor nodes in the scenario. This error basically evaluated how different the obtained curated data series $x_{i}^{*}\left[n\right]$ was from the original information generated by sensor nodes $y_{i}\left[n\right]$. To improve the clarity in the results, this MSE was expressed as a percentage (74). The same experiment was performed for the two proposed operation modes: offline data curation and real-time data curation. Furthermore, the proposed experiment was repeated for different relative lengths of the expanded time interval $\left[n_{1}^{e},\;n_{2}^{e}\right]$, with respect to the curation interval $\left[n_{1},\;n_{2}\right]$ (72). All sensors were generating data every 30 s.


(73) $$MSE=\frac{1}{N\cdot \left(n_{2}-n_{1}\right)}\overset{N}{\underset{i=1}{\sum }}\overset{n_{2}}{\underset{n=n_{1}}{\sum }}{\left(x_{i}^{*}\left[n\right]-y_{i}\left[n\right]\right)}^{2}$$

(74) $$MSE\;\left(\%\right)=\frac{MSE}{\frac{1}{N\cdot \left(n_{2}-n_{1}\right)}\sum_{i=1}^{N}\sum_{n=n_{1}}^{n_{2}}{\left(y_{i}\left[n\right]\right)}^{2}}\;\cdot 100$$

Furthermore, to evaluate the improvement provided by the proposed scheme compared to existing mechanisms, results in the third experiment were compared to results reported by state-of-the-art solutions [56]. Specifically, to perform a coherent and valid comparison, we selected a low-cost, error-correction framework for wireless sensor networks. This scenario was similar to Industry 4.0 applications, and the selected solution also included a prediction and a correction phase, so the mechanisms were technically comparable, so results could be also compared. Results for this state-of-the-art solution [56] were obtained through a secondary simulation scenario with the same characteristics and setup employed for the proposed new approach.

**Figure 2:**
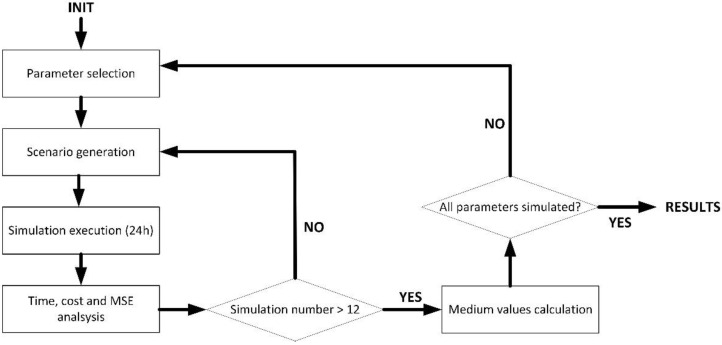
Experimental simulation setup.

For these three initial experiments, a simulation scenario was built and run using the MATLAB 2019B suite. The simulation scenario represented a chemical industry where environmental data are collected to make decisions about how safe it is to work on the pending activities. Four different kinds of sensor nodes were considered: temperature sensors, humidity sensors, CO_2_ sensors (air quality) and volatile compounds sensors (detecting dangerous gas emissions). The scenario included a random composition, using all these four types of sensor nodes. All sensors generated data every $T_{i}$ seconds, although they were not synchronized. Each sensor started operating at a random instant within the first minute of the system running. Simulated sensor nodes were designed to represent an ESP-32 microprocessor (with a 12-bit ADC and a 16-bit architecture). Figure 2 shows the experimental simulation setup.

The simulated scenario was based on a minimum-sized industry, where distances were never larger than 50 m. The decision-making software module collected information from sensor nodes using LoRaWAN wireless technology. The global environment was suburban, so the level of interferences was moderate-low.

Malfunctions were represented by models and functions included in the MATLAB libraries, so we guaranteed the independence of the system configuration phase and the system evaluation phase. In that way, results were more relevant. Models for jitter were introduced from Simulink and included duty-cycle-distortion deterministic jitter, random jitter, sinusoidal jitter and noise. In this work, only deterministic and random jitter were considered. The different types of jitter were injected into devices according to the IBIS-AMI specifications [57]. Delays were managed through the Control System Toolbox, which allowed integrating the input delay, the output delay and the independent transport delays in the transfer function and the frequency-response data. In this work, we are using first-order plus dead time models and state-space models with input and output delays. Regarding BER, MATLAB includes the Bit Error Rate Analysis App Environment, which can integrate different instances of the numerical models generated through a Monte Carlo Analysis Simulink block and simulation. Finally, numerical errors were introduced and modeled as numerical noise in devices, using the Simulink framework and the IBIS-AMI specifications (in a similar way as described and conducted for jitter models).

Simulations represented a total of 24 h of system operation. To remove the impact of exogenous variables in the results, each simulation was repeated 12 times. Results were calculated as the mean value of all these simulations for each system configuration. All simulations were performed using a Linux architecture (Ubuntu 20.04 LTS) with the following hardware characteristics: Dell R540 Rack 2U, 96 GB RAM, two processors (Intel Xeon Silver 4114 2.2 GB, HD 2 TB SATA 7.2K rpm). Simulations were performed under isolation conditions: Only the MATLAB 2019B suite was installed and running in the machine; all other services, software or internet connection were stopped or removed. The objective was to remove as much as possible all validity threats. The global simulation time was variable and automatically calculated by the system to get data representing 24 h of system operation.

The fourth and final experiment also aimed to evaluate the precision and curation success of the proposed predictive-correction algorithm. However, in the last experiment, we employed a real hardware platform. In this case, in an emulation environment representing the referred chemical industry, four nodes (one of each type) were deployed together with a LoRaWAN gateway. The sensor nodes configuration was identical to the proposed configuration for the simulation scenarios. In particular, all sensors were generating data every 30 s. In this case, measures about the proposal’s precision were obtained as the mean square error (73)–(74) as well. The experiment was also performed for the two proposed operation modes: offline data curation and real-time data curation. The proposed experiment was repeated for different relative lengths of the expanded time interval $\left[n_{1}^{e},n_{2}^{e}\;\right]$ with respect to the curation interval $\left[n_{1},n_{2}\right]$ as performed in the third experiment. In this case, malfunctions were introduced by actual environmental and technological factors. For each configuration, the system was operating 24 h (so results in the third and fourth experiments are comparable).

For all these experiments, the configuration for the proposed prediction-correction algorithm is shown in Table 2.

### 4.2. Results

**Figure 3:**
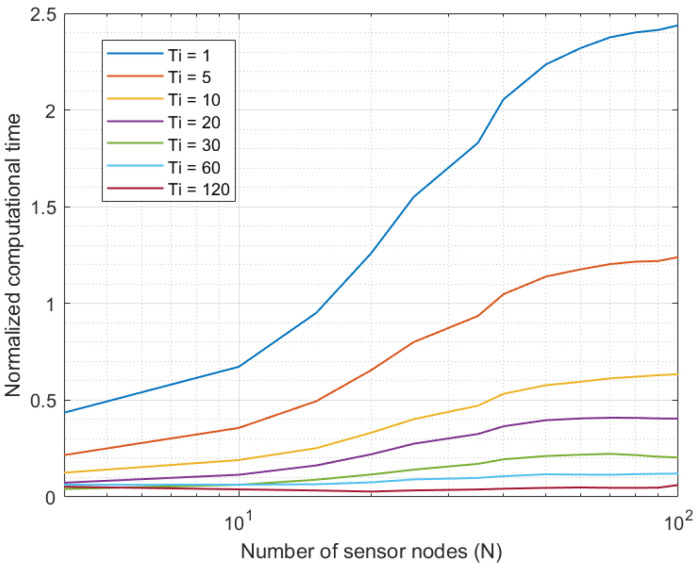
Results of the first experiment.

Figure 3 shows the results of the first experiment. As can be seen, for all Industry 4.0 systems up to 10 sensor nodes, the proposed solution was able to curate all data series in real time, as the computational time was below the sampling period. However, systems with higher sample-generation rates ($T_{i}=1$ s and $T_{i}=5$ s) got congested when the number of nodes went up. Specifically, systems with 20 and 40 elements, respectively, caused the system to become unavailable. Any other higher sampling period did not cause congestion in the system, even with platforms including up to 100 sensor nodes.

Although for higher device densities and higher amounts of sensor nodes, the proposed system may get congested with higher sampling periods, 100 nodes is above the number of nodes most current Industry 4.0 platforms include.

**Figure 4:**
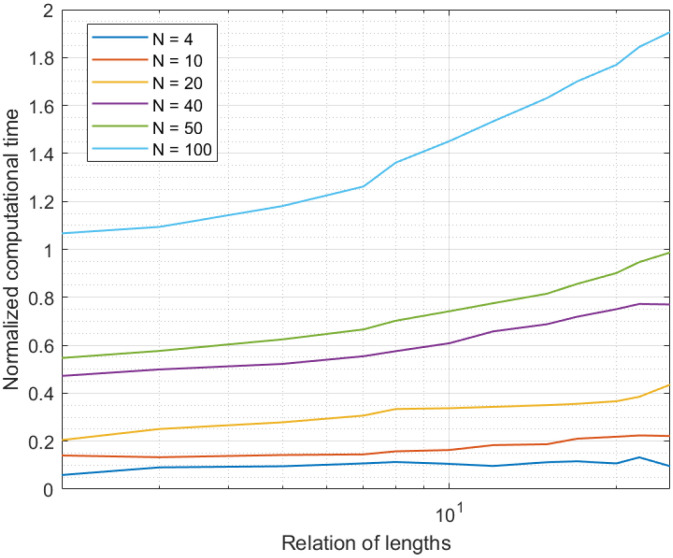
Results of the second experiment.

Figure 4 shows the results of the second experiment. In this case, we evaluated the computational cost in the offline data curation. As can be seen, in only one case was the computational time above the sampling period: for networks with 100 sensor nodes. This situation was caused by a very populated network (100 sensor nodes), where data were generated at a high rate; greater than the processing rate offered by the prediction-correction mechanism (the data generation rate went up when the number of nodes in the network increased). Thus, after a few samples, the system did not process data “on the fly” and the new data were queued. Therefore, long term, the medium computational time was higher than the sampling period, because of data queues and the congestion caused by the high data-generation rate. In any case, in general and as said before, 101 is a number of devices above the current needs of common Industry 4.0 deployments (although future pervasive platforms may go beyond this number).

Only a second system configuration may cause the system to get congested: for an Industry 4.0 system with 50 devices and an extended time interval 25 times higher than the curation interval. Although this is a large number of samples to process, the reduced complexity of the proposed solution allowed (even in this case) computational times slightly below the sampling period.

As a conclusion of these two initial experiments, the proposed solution successfully operated in real time and offline. Besides, it is scalable to large systems, above the current Industry 4.0 needs. However, for future pervasive sensing platforms, the proposed solution may require powerful cloud systems or the definition of a distributed or edge computing scheme.

**Figure 5:**
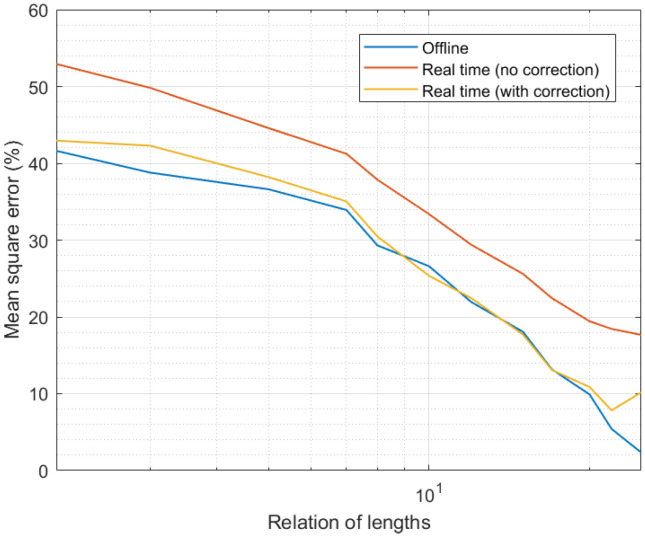
Results of the third experiment.

Figure 5 presents the results of the third experiment. As can be seen, in all situations the MSE was below 50%, even for weak configurations, such as expanded time intervals that were only less than two times higher than the curation interval. As the number of samples that were integrated into the candidate calculation process went up, the MSE clearly went down. For configurations where the length of the expanded interval was around 25 times the length of the curation interval, the MSE was almost null, and the remaining error may be considered residual and intrinsic to all processing schemes.

It was also interesting to analyze the impact of the proposed correction phase. As can be seen, real-time curation showed worse performance than offline data curation, as the available information was more reduced. The MSE may be between 10% and 20% higher in real-time data curation than in offline curation for the same system configuration. However, when considering the proposed correction phase, which was able to integrate future information in the previously obtained curated data series, real-time data curation may reach the same precision as offline mechanisms.

**Figure 6:**
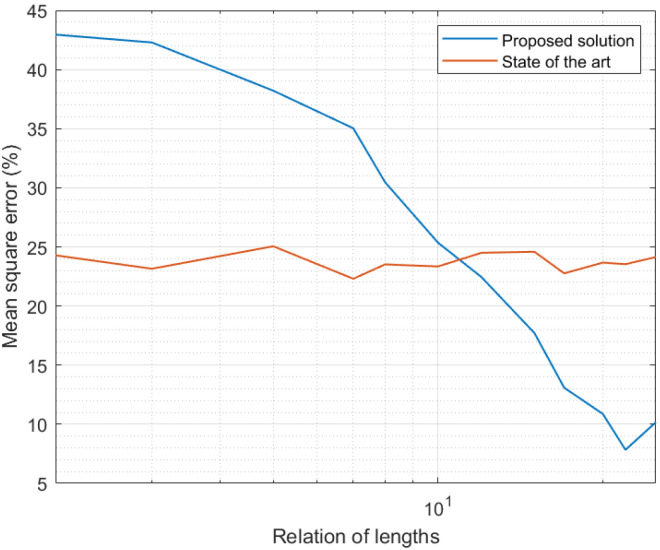
Results of the third experiment: comparison with state-of-the-art solutions [56].

Figure 6 shows a comparison between results obtained in the third experiment and results reported by state-of-the-art solutions [56]. As state-of-the-art mechanisms do not depend on the length of the expanded time interval (this notion was defined only in the new proposed scheme), the MSE for the previously reported solution was constant. Small variations displayed in Figure 6 were caused by numerical effects in the simulation. As can be seen, for expanded time intervals very similar in length to the curation interval, the MSE was lower in state-of-the-art solutions. Decision trees in state-of-the-art mechanisms, to be trained, analyze large amounts of data, so their models are much more precise than the models generated in the proposed framework. However, as larger expanded intervals were considered, the MSE in the proposed solution was greatly reduced, while state-of-the-art mechanisms remained constant. In fact, for relation of lengths above 20, the proposed solution showed an MSE 50% lower than the previously reported mechanism. In conclusion, for models obtained from the analysis of comparable amounts of samples, the proposed framework produced curated data series 50% more precise.

If we evaluate together Figure 3, Figure 4, Figure 5 and Figure 6, we can conclude the proposed solution successfully obtained a precise curated data series in Industry 4.0 platforms. Besides, if we assume an error around 5%, the proposed algorithm may be deployed in scenarios with a large number of sensor nodes, above the current state of the art in Industry 4.0 solutions.

**Figure 7:**
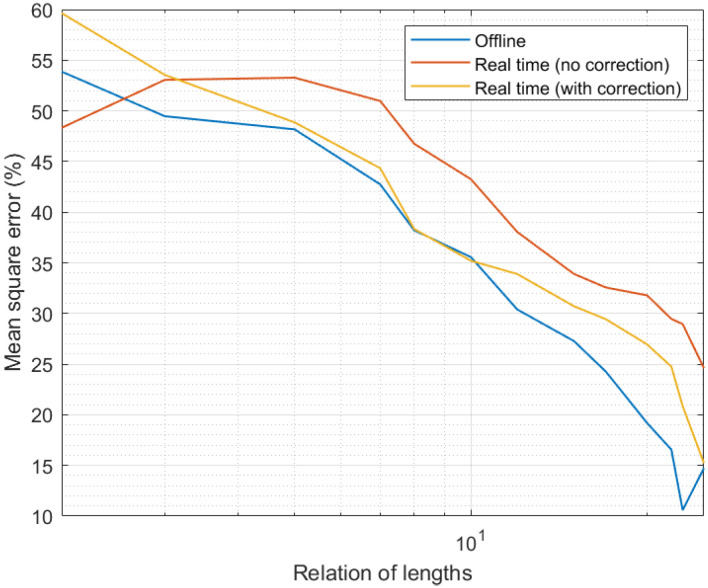
Results of the fourth experiment.

Finally, Figure 7 shows the results of the fourth experiment. As can be seen, the evolution of the MSE with the relative interval length was similar to the one observed in Figure 4 (third experiment). As larger intervals were considered and more samples introduced in the candidate calculation process, the precision went up and the MSE went down. However, in this case, the precision was slightly lower than in the simulation study; in particular, we can see the MSE was around 5% higher. In any case, the qualitative evolution of the proposed mechanism was equivalent in simulation and real scenarios. Only in one situation was the performance different in a relevant manner: for curation and expanded time intervals with the same length (relation of lengths equal to the unit). Under those circumstances, in simulation scenarios, the correction phase had a smooth behavior and it still reduced the MSE. However, in real scenarios, when the relation of lengths was equal to the unit, not enough information was provided to the correction algorithm and thresholds did not converge properly. As a consequence, the correction phase introduced an additional error instead of improving the data quality. This situation, in any case, was very transitory and solved immediately when the expanded interval was even slightly higher than the curation interval (relation of lengths above the unit). Despite this fact, and once more, the impact of the correction phase was relevant, even more in this real hardware implementation (MSE may grow up to around 90% without the correction phase).

Thus, results allowed us to conclude the proposed solution was successful and valid as a data curation technology for Industry 4.0, focused on improving sensor interoperability.

## 5. Conclusions

At the software level, real-time algorithms and automatic decision-making mechanisms are very sensitive to the quality of received data. Common malfunctions in sensor nodes, such as delays, numerical errors, corrupted data or inactivity periods, may cause a critical problem if an inadequate decision is made based on those data. The most common solution to this problem is the adaptation and transformation of high-level software components to tolerate these effects, but this calibration turns interoperability between physical sensors and software modules into a very problematic issue.

Therefore, new solutions that guarantee the interoperability of all sensors with the software elements in Industry 4.0 solutions are needed. In this paper, we proposed a solution based on numerical algorithms following a predictor-corrector architecture. Using a combination of techniques, such as Lagrange polynomial and Hermite interpolation, data series may be adapted to the requirements of Industry 4.0 software algorithms. Series may be expanded, contracted or completed using predicted samples, which are later updated and corrected using the real information (if received).

Through this process, the resulting curated time series has enough quality to be employed with any software module (artificial intelligence, decision making, etc.), guaranteeing the interoperability of all sensor nodes with the high-level applications (which now do not require any adaptation or calibration procedure).

Results show the proposed solution successfully operated in real time and offline and it was scalable to large systems, above the current Industry 4.0 needs. Besides, we can conclude the proposed solution successfully obtained a precise curated data series in Industry 4.0 platforms, even in scenarios with large number of sensor nodes.

Future works will consider the proposed solution in large Industry 4.0 deployments with intense industrial activity, where the environment is more hostile and the operation conditions are more critical.

## Figures and Tables

**Table 1:** Most relevant symbols and variables.

Parameter	Meaning	Parameter	Meaning
T	Industry 4.0 platform	$S_{i}$	Sensor node
N	Number of data flows	$\lambda_{l}$	Malfunction function
L	Number of malfunctions	$x_{i}\left[n\right]$	Original data series
Ω	Universe of values for data series	$f_{s}^{i}$	Sampling frequency
$n_{0}$	Current time instant	$n_{1}^{e}$	Limit in the expanded time interval
$x_{i}^{*}\left[n\right]$	Curated time series	$p_{mal}$	Probability of malfunction
${𝔵}_{c}$	Candidate to curated data series	$d_{g}$	Distance between distributions
$p_{rx}$	Probability of data in reception	$y_{i}\left[n\right]$	Real data generated by physical sensors
C	Number of candidates	$p_{i}$	Probability of original data
$I_{\Omega }$	Identity function	$B_{s}$	Stationary time interval
$\Pi_{\Omega }$	Partition of the universe Ω	Δ	Number of elements in a partition

**Table 2:** Proposed algorithm configuration.

Parameter	Value	Comments
$E_{b}$	0.2 dBm	Typical value according to the hardware capabilities of ESP-32 microprocessor
$\gamma_{lim}$	4	Standard value for the correction capacity of cyclic codes
$\Lambda_{max}$	$2^{16}$	Associated to a 16-bit architecture
Σ	4096	Associated to a 12-bit ADC
Γ	1 h	Standard value in traffic theory
β	3	Typical mathematical order for high-precision applications
